# Clinical Outcomes of Surgical–Orthodontic Management of Impacted Mandibular Canines: A Systematic Review

**DOI:** 10.3390/dj14100632

**Published:** 2026-10-02

**Authors:** Francesca Stramandinoli, Silvia Mura, Maurizio Ledda, Benedetta Salis, Beatrice Tandurella, Nerea Frances Garcia, Teresa Cobo, Alessio Verdecchia, Enrico Spinas

**Affiliations:** 1 Department of Surgical Sciences, Postgraduate School in Orthodontics, University of Cagliari, 09124 Cagliari, Italy; 2 Orthodontics Division, Instituto Asturiano de Odontología, Universidad de Oviedo, 33006 Oviedo, Spain

**Keywords:** orthodontics, impacted mandibular canine, orthodontic traction, surgical exposure, transmigrated mandibular canine, surgical–orthodontic treatment

## Abstract

**Background**: Impaction of mandibular canines is an uncommon clinical condition, and transmigration represents a severe positional variant within this spectrum. In contrast to maxillary canine impaction, the management of impacted mandibular canines remains less standardized and largely experience-based. This systematic review aimed to critically evaluate the clinical outcomes of surgical exposure followed by orthodontic traction in impacted mandibular canines. **Methods**: A systematic search was conducted across five electronic databases (PubMed, Web of Science, Scopus, Embase, and Cochrane Library) up to February 2026. A total of 1512 records were identified; after duplicate removal, 1307 studies underwent title and abstract screening. Fifteen full-text articles were assessed for eligibility, and four retrospective analytical studies met the predefined inclusion criteria. The primary outcome was successful orthodontic alignment. Secondary outcomes included treatment duration and reported complications. Risk of bias was assessed using ROBINS-I, and certainty of evidence was evaluated according to the GRADE approach. **Results**: Success rates ranged from 57% to 100% across studies. Overall, 31/42 canines (73.8%) were reported as successfully aligned; however, heterogeneity in case selection, outcome definitions, and reporting precluded quantitative synthesis. In most studies, success was defined primarily as positional alignment within the dental arch, with limited or absent assessment of periodontal status, pulp vitality, functional integration, or long-term stability. All included studies were judged to have a serious overall risk of bias. According to GRADE, the certainty of evidence for all evaluated outcomes was very low. **Conclusions**: Surgical exposure followed by orthodontic traction may represent a feasible option in carefully selected cases of impacted mandibular canines, particularly in younger patients and in the absence of severe displacement. However, given the serious risk of bias, small cumulative sample size, and very low certainty of evidence, these estimates must be interpreted with extreme caution. Unlike maxillary canine impaction, the current evidence base does not support protocol-driven management for mandibular cases, and clinical decision-making remains primarily individualized.

## 1. Introduction

The permanent mandibular canine plays a crucial role in occlusal function, arch stability, and facial aesthetics. Despite its clinical importance, mandibular canine impaction remains considerably less prevalent than maxillary canine impaction, with epidemiological studies consistently reporting a markedly lower incidence in the mandible [1,2,3,4]. The lower frequency of this condition has likely contributed not only to reduced scientific attention but also to a qualitative disparity in the level of methodological rigor supporting diagnosis and treatment.

Maxillary canine impaction has been extensively investigated over several decades. Radiographic predictors of eruption disturbance, sector-based positional analyses, angulation thresholds, interceptive extraction strategies, and refined biomechanical protocols have been systematically explored in analytical and prospective investigations [5,6]. As a result, treatment planning for impacted maxillary canines is increasingly supported by structured diagnostic frameworks and reproducible clinical algorithms. In contrast, the literature addressing impacted mandibular canines remains limited and methodologically heterogeneous. Clear prognostic indicators have not been validated, and no evidence-based algorithm comparable to those proposed for maxillary impaction has been established. Consequently, clinical decision-making in mandibular cases frequently relies on individualized assessment and operator experience rather than on robust predictive models.

Within the spectrum of mandibular impaction, transmigration represents a particularly complex positional presentation. Originally defined by Javid (1985) as the pre-eruptive migration of a mandibular canine across the midline when more than half of the tooth length crosses the mandibular symphysis, transmigration was subsequently classified into five positional patterns by Mupparapu (2002) [7,8]. While this classification provides a descriptive radiographic framework, it does not establish prognostic thresholds nor standardized therapeutic indications. Furthermore, most available data on transmigrated canines derive from isolated case reports or small case series, limiting the possibility of drawing reproducible conclusions regarding treatment feasibility or long-term stability.

Previous reviews have summarized management approaches and case experiences; however, many have included case reports and heterogeneous study designs without applying structured risk-of-bias assessment or certainty-of-evidence grading. To date, a systematic synthesis focusing exclusively on analytical studies reporting extractable outcome data for surgical exposure followed by orthodontic traction in impacted mandibular canines remains lacking. In light of these limitations, a structured and methodologically rigorous synthesis of the available evidence is required. The primary aim of the present systematic review was to evaluate the clinical outcomes of surgical exposure followed by orthodontic traction in impacted mandibular canines. Secondary aims were to evaluate treatment-related variables and to assess the methodological quality and certainty of the available evidence.

## 2. Materials and Methods

### 2.1. Protocol and Registration

The present systematic review was conducted in accordance with the Preferred Reporting Items for Systematic Reviews and Meta-Analyses (PRISMA 2020) statement [9,10]. Protocol development and registration were informed by PRISMA-P recommendations and current Cochrane methodological guidance [11,12]. The review protocol was prospectively registered in PROSPERO, the International Prospective Register of Systematic Reviews, on 25 January 2026 (Registration number: CRD420261289636). The completed PRISMA 2020 checklist is provided as Supplementary Materials.

### 2.2. Search Strategy

The search covered all articles published from database inception to 19 February 2026 in the following electronic databases: PubMed, Web of Science, Scopus, Embase, and the Cochrane Library. No restrictions regarding language or publication date were applied. The search strategy was adapted to the specific syntax of each database, and the detailed search strings are reported in Table 1. All retrieved records were imported into reference management software (Zotero^®^, version 7.0.32, Corporation for Digital Scholarship, Vienna, VA, USA) for automated duplicate detection, followed by manual screening to confirm duplicate removal. Grey literature was explored through manual screening of the reference lists of all included studies to identify potentially relevant publications not captured through electronic database searching. Reporting of the electronic search strategy was structured according to PRISMA-S recommendations [13].

### 2.3. Eligibility Criteria

Eligibility criteria were defined according to the PICO framework (Population, Intervention, Comparison, Outcome), as summarized in Table 2.

Randomized controlled trials, prospective cohort studies, retrospective cohort studies, and case–control studies conducted on human subjects were considered eligible. Although randomized trials were eligible a priori, none meeting the inclusion criteria were identified. Case reports, case series, reviews, meta-analyses, editorials, and conference abstracts were excluded to ensure inclusion of analytical study designs providing extractable outcome data. Studies evaluating only surgical extraction, autotransplantation, observation, or purely diagnostic approaches without reporting outcomes of surgical–orthodontic alignment were also excluded. In vitro and animal studies were not eligible. Studies lacking clearly extractable data regarding orthodontic alignment outcomes or not aligned with the predefined PICO framework were excluded. Articles for which the full text could not be retrieved despite extensive search efforts were also excluded.

### 2.4. Data Collection

Two authors (F.S. and M.L.) independently carried out the research process first, followed by the screening of the obtained results. To assess the level of agreement among the reviewers, Cohen’s kappa coefficient was calculated [14]. In case of disagreement during the data extraction process, a third reviewer (A.V.) participated in the discussion to reach a decision. The analysis showed a substantial agreement among the authors (Cohen’s kappa: 0.70). The selection process was performed in two stages: first, all potentially eligible studies were screened by reading titles and abstracts. Second screening was performed according to predefined inclusion and exclusion criteria by full-text analysis of the articles. The collected data were organized into thematic sections to provide a clear and comprehensive overview of the included studies. Specifically, the data were grouped as follows:

- Study characteristics, including author, year of publication, country, study design, sample size, age range/mean age;
- Clinical and prognostic characteristics of treated cases, including the number of mandibular canines managed with surgical exposure followed by orthodontic traction, age data for the mandibular subgroup, presence of transmigration when reported, and orthodontic mechanics employed;
- Primary and secondary clinical outcomes, including successful alignment, failure rate, extractions during treatment, and reported complications.

Outcomes were consistently recorded at the tooth level, as reported in the original studies.

### 2.5. Quality Assessment

The methodological quality of the included studies was evaluated using the Risk Of Bias In Non-randomized Studies of Interventions (ROBINS-I) tool [15]. This instrument assesses bias across seven domains: (1) bias due to confounding; (2) bias in selection of participants; (3) bias in classification of interventions; (4) bias due to deviations from intended interventions; (5) bias due to missing data; (6) bias in measurement of outcomes; and (7) bias in selection of the reported result. Each domain was judged as low, moderate, serious, critical risk of bias, or no information, according to the guidance provided by the ROBINS-I framework. Two reviewers (F.S. and M.L.) independently assessed each study, and disagreements were resolved through discussion and consensus, with involvement of a third reviewer (E.S.) when necessary. Overall risk of bias was determined according to the worst-domain approach, as recommended by the ROBINS-I guidance.

The certainty of evidence for each main outcome was evaluated using the Grading of Recommendations Assessment, Development and Evaluation (GRADE) approach [16,17,18]. The GRADE framework considers risk of bias, inconsistency, indirectness, imprecision, and publication bias to classify the certainty of evidence as high, moderate, low, or very low. Given the observational nature of the included studies, evidence was initially rated as low and downgraded when appropriate based on predefined criteria If randomized controlled trials had been included, risk of bias would have been assessed using the revised Cochrane RoB 2 tool [19].

## 3. Results

### 3.1. Screening and Selection Process

**Figure 1:**
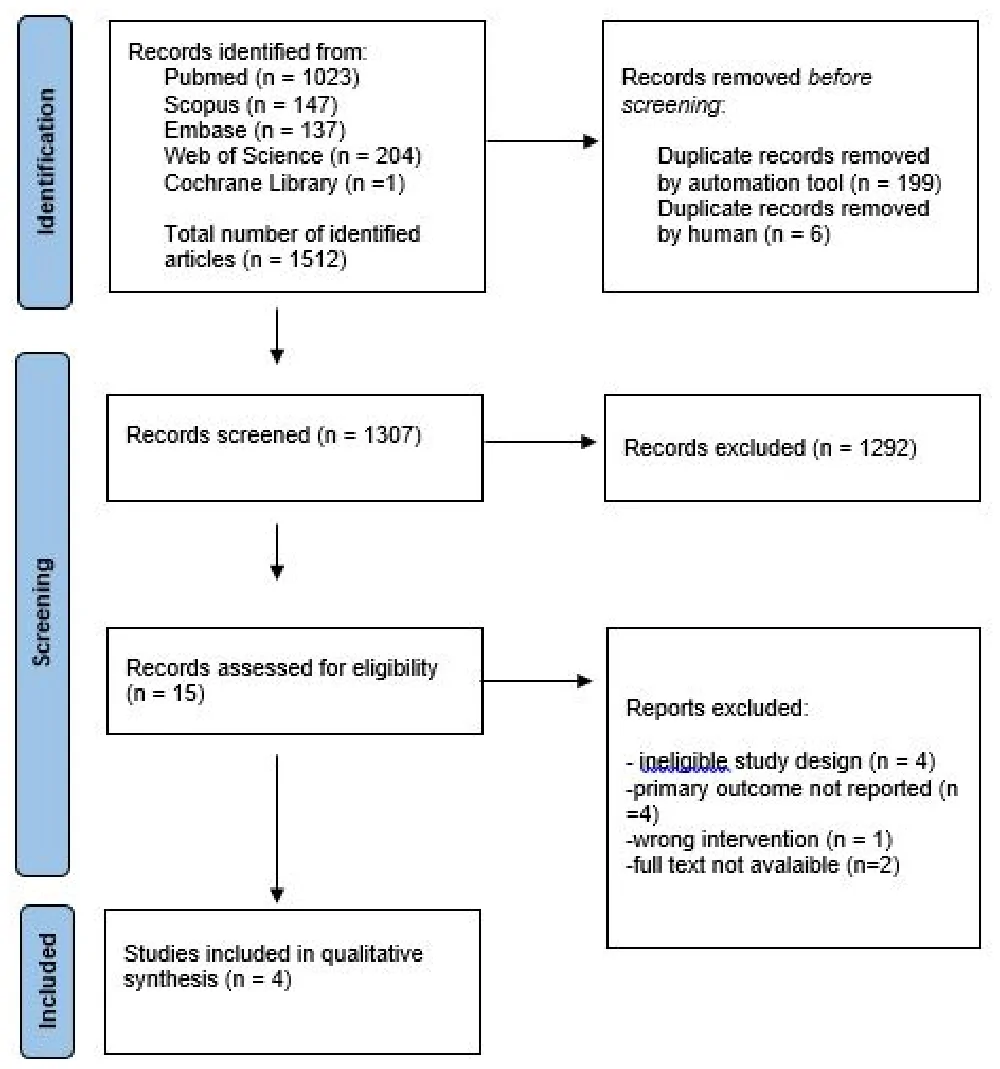
PRISMA flowchart: flow diagram of the performed search.

The search strategy yielded a total of 1512 articles through the consultation of five major databases: PubMed, Web of Science, Scopus, Embase, and the Cochrane Library. Duplicates were initially removed using reference management software, resulting in 1313 records, followed by a manual deduplication process that excluded an additional 6 duplicates, leaving 1307 unique records. The remaining 1307 articles were screened based on title and abstract, leading to the exclusion of 1292 articles that did not meet the predefined eligibility criteria. Fifteen studies were therefore selected for full-text assessment. During the full-text evaluation phase, eleven articles were excluded for the following reasons: ineligible study design (case reports, case series, reviews), purely diagnostic or epidemiological focus without assessment of treatment outcomes, lack of extractable clinical data, non-alignment with the predefined PICO framework, or unavailability of the full text despite extensive search efforts. At the end of the selection process, four observational analytical studies met the predefined inclusion and exclusion criteria and were included in the qualitative synthesis. The study selection process is summarized in the PRISMA flow diagram (Figure 1).

### 3.2. General Characteristics of the Selected Studies

The electronic search was not restricted by publication date; the four studies that met the inclusion criteria were published between 2011 and 2021.

Although randomized controlled trials were considered eligible a priori, no RCTs meeting the predefined inclusion criteria were identified. All included investigations were observational in nature and retrospective in design. Across the four included studies, a total of 183 patients presenting with impacted mandibular canines were analyzed. Not all patients underwent surgical exposure followed by orthodontic traction, as alternative management strategies were reported in the included studies. Sample sizes varied between studies, ranging from 21 to 74 patients. In several investigations, bilateral impaction was reported, resulting in a higher number of impacted teeth than patients. In studies that included both maxillary and mandibular impacted canines, only the subgroup of patients presenting with mandibular impaction was extracted and considered for the present analysis. This methodological approach ensured strict adherence to the predefined PICO framework and prevented overestimation of the study population by excluding non-mandibular cases. The geographical distribution of the included studies demonstrates international representation. Early contributions originated from Turkey [20], followed by investigations conducted in Hong Kong, China [21], Austria [22], and Poland [23]. This distribution reflects the limited but globally dispersed body of evidence regarding the management of impacted mandibular canines. The included studies reported different management strategies for impacted mandibular canines, among which surgical exposure followed by orthodontic traction represented one of the evaluated treatment approaches. Although different surgical techniques and orthodontic mechanics were employed across studies, all investigations assessed the feasibility and clinical success of surgical–orthodontic alignment.

Table 3 summarizes the general characteristics of the four studies included in this systematic review.

### 3.3. Clinical Characteristics of Mandibular Canines Treated with Surgical Exposure and Orthodontic Traction

This section summarizes the clinical characteristics of mandibular canines treated with surgical exposure followed by orthodontic traction, in accordance with the predefined PICO criteria. Only cases undergoing this combined surgical–orthodontic approach were included. Across the four included retrospective studies, a total of 42 impacted mandibular canines were managed with surgical exposure followed by orthodontic traction. These teeth represent the subset of cases within the overall study populations that underwent surgical exposure followed by orthodontic traction. Outcomes were consistently reported at the tooth level. Age data were not consistently stratified for the specific subset of teeth treated with traction, limiting the ability to assess age as an independent prognostic factor. The reported mean age values varied across studies. The presence of transmigrated mandibular canines was reported in three of the four studies; however, the exact number of transmigrated teeth treated with surgical exposure and orthodontic traction was not consistently specified, precluding subgroup analysis. With regard to orthodontic mechanics, three studies described the use of conventional fixed-appliance traction following surgical exposure, while one study reported the use of temporary anchorage devices (TADs) to assist traction.

The clinical and treatment-related characteristics extracted from the included studies are summarized in Table 4.

### 3.4. Primary and Secondary Outcomes

The primary outcome was the successful orthodontic alignment of the impacted mandibular canine, while secondary outcomes included treatment discontinuation, extractions during treatment, and reported complications. Due to heterogeneity in study design and outcome reporting, not all variables were consistently available across the included studies. Outcome reporting varied considerably, with some authors providing quantitative data on treatment success and others offering limited information on secondary outcomes. The primary outcome was explicitly reported in all four included retrospective studies [20,21,22,23]. However, definitions of success and reporting formats were not entirely uniform, and outcomes were consistently presented at the tooth level. Aras et al. reported a success rate of 69.6% (16 out of 23 canines). Sajnani & King observed a success rate of 57.1% (4 out of 7 canines), with failures occurring following lack of orthodontic movement. Sinko et al. reported a 75.0% success rate (3 out of 4 canines), while Stabryła et al. documented successful alignment in all treated cases (8 out of 8 canines; 100.0%). Across the included studies, 31 out of 42 treated mandibular canines were reported as successfully aligned. Due to clinical and methodological heterogeneity, no quantitative synthesis was performed. Importantly, the operational definition of “success” differed across studies [20,21,22,23]. In most investigations, success was defined solely as radiographic or clinical alignment of the canine within the dental arch, without systematic evaluation of periodontal health, pulp vitality, occlusal function, or long-term stability [20,21,22]. No study incorporated standardized composite outcome measures. This heterogeneity limits comparability across studies and may lead to overestimation of favorable outcomes. Secondary outcomes were inconsistently described across studies. Extractions during treatment were reported in two investigations, while treatment discontinuation was documented in one study. Root resorption and ankylosis were mentioned in a single study; other investigations did not systematically assess or report these complications. Information on treatment duration was not uniformly available and was not consistently stratified for the treated subgroup, precluding meaningful comparison across studies. Table 5 provides a comprehensive summary of the extracted primary and secondary outcomes across the included investigations.

### 3.5. Risk of Bias Assessment

**Figure 2:**
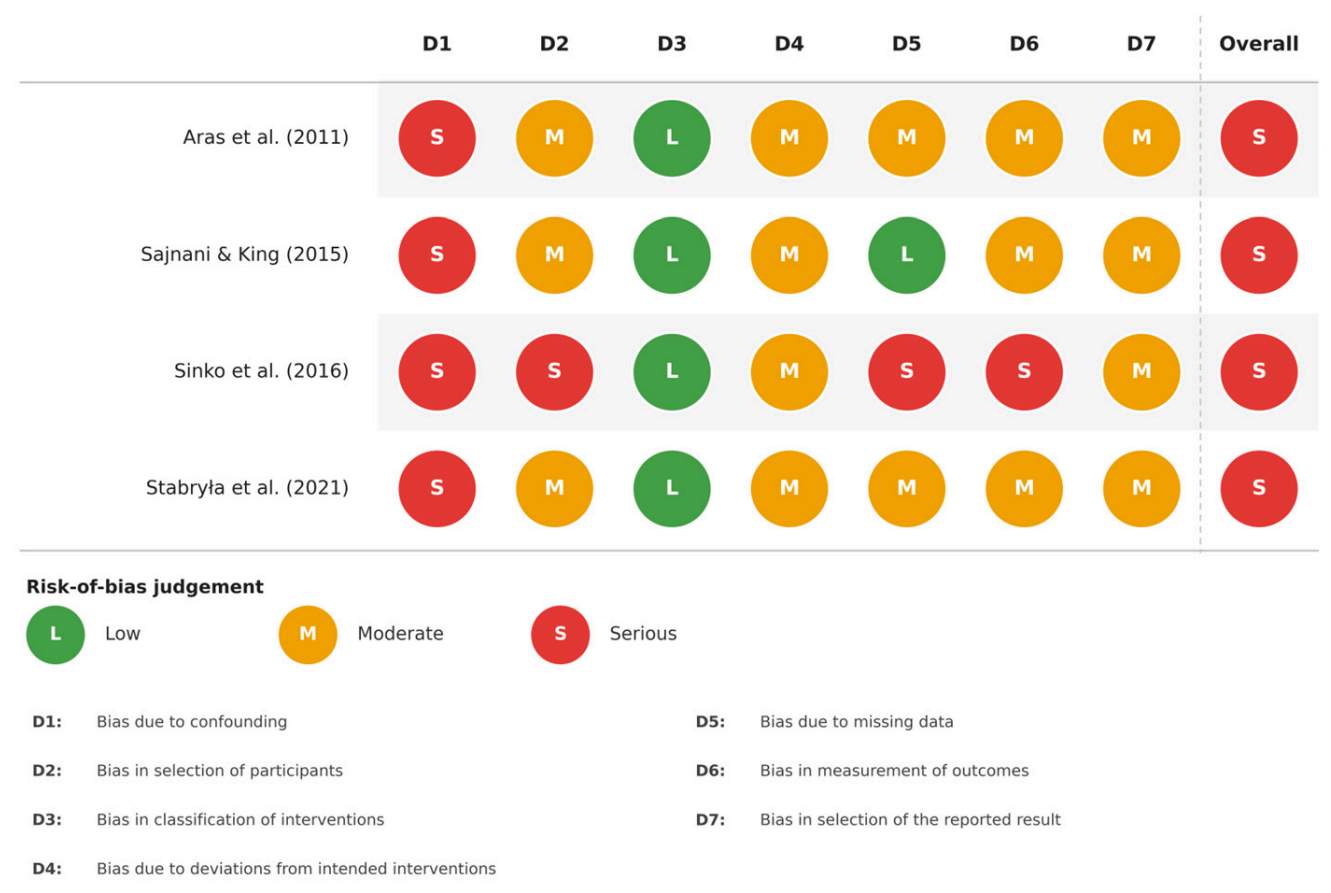
Risk of bias of the included studies [20,21,22,23] according to the Risk of Bias In Non-randomized Studies of Interventions (ROBINS-I).

All included studies were rated as having a serious overall risk of bias according to the ROBINS-I tool, in accordance with the worst-domain rule. The elevated overall risk of bias was mainly driven by confounding (D1) and, in some studies, by limitations in the selection of participants (D2), which are inherent to retrospective observational designs and small sample sizes. A summary of the risk-of-bias judgments across the ROBINS-I domains is presented in Figure 2. Although the overall risk of bias was judged as serious for all included studies, several domains were rated as low or moderate risk in individual investigations. In particular, bias in the classification of interventions (D3) was judged as low, as surgical exposure followed by orthodontic traction was clearly defined and consistently identifiable across studies. Bias due to missing data (D5) was considered low in studies in which the primary outcome was available for all included patients or teeth, while it was rated as moderate or serious when outcome reporting was incomplete. Similarly, bias in the measurement of outcomes (D6) was judged as moderate in studies reporting clearly defined and consistently assessed treatment success, and serious when outcome definitions were unclear or embedded within multimodal treatment strategies. The serious overall risk of bias observed across studies primarily reflects uncontrolled confounding, retrospective treatment allocation, and limited control over selection criteria. These methodological constraints may have influenced both case selection and outcome reporting, thereby limiting internal validity and reducing confidence in the reported effect estimates. Given the retrospective nature of the included studies and the absence of blinded outcome assessment, bias in outcome measurement (Domain 6) cannot be excluded.

### 3.6. Quality of Evidence

The certainty of evidence for each main outcome was evaluated using the Grading of Recommendations Assessment, Development and Evaluation (GRADE) approach. As all included studies were observational in design, the certainty of evidence was initially rated as low. Downgrading was subsequently applied based on predefined criteria including risk of bias, inconsistency, indirectness, imprecision, and potential publication bias. For the primary outcome (successful orthodontic alignment), evidence was downgraded due to serious risk of bias (ROBINS-I overall serious rating in all included studies), inconsistency in reported success rates (ranging from 57% to 100%), and imprecision related to the small cumulative sample size (*n* = 42 treated teeth) and absence of pooled quantitative estimates. The overall certainty of evidence was therefore judged as very low. Similarly, secondary outcomes—including treatment duration, failure of orthodontic movement, root resorption, ankylosis, periodontal outcomes, and outcomes in transmigrated canines—were characterized by sparse data, non-standardized reporting, and limited stratification. These limitations resulted in further downgrading for imprecision and inconsistency. No outcome reached a certainty level above very low. Very low certainty indicates that the true effect of surgical exposure followed by orthodontic traction in impacted mandibular canines is likely to be substantially different from the estimates reported in the available literature. A detailed summary of findings and GRADE ratings is presented in Table 6.

## 4. Discussion

This systematic review demonstrates that the current evidence regarding surgical exposure followed by orthodontic traction in impacted mandibular canines is quantitatively limited and methodologically fragile. Importantly, this limitation is not merely numerical but reflects a broader lack of analytical and hypothesis-driven research in this field. Although preservation of the natural tooth remains a desirable clinical objective, the available data do not allow the development of reproducible prognostic models or standardized therapeutic algorithms. Consequently, any apparent clinical success should be interpreted within the context of substantial uncertainty. The discrepancy between mandibular and maxillary canine impaction is not merely epidemiological but methodological. While maxillary impaction has benefited from progressively refined radiographic predictors, positional classifications, and structured biomechanical protocols supported by analytical and prospective investigations, mandibular impaction remains primarily investigated through small retrospective cohorts [5,6,20,21,22,23]. Consequently, treatment planning in mandibular cases lacks the algorithm-driven framework increasingly available for maxillary canines. This disparity ultimately translates into a lower level of biological and clinical predictability in mandibular cases. The rarity of mandibular canine impaction likely contributes substantially to this disparity. Epidemiological studies consistently report a markedly lower prevalence compared with maxillary impaction [1,2,3,4]. Recruitment periods extending over more than a decade in the included studies [20,21,22] indirectly reflect the low incidence of the condition. Such prolonged recruitment timelines also increase the risk of heterogeneity in diagnostic criteria, imaging techniques, and treatment protocols over time. Such structural limitations inherently restrict the feasibility of large prospective or randomized investigations, thereby constraining the strength and generalizability of available conclusions. Within this context, surgical exposure followed by orthodontic traction represents only one of several management options. Extraction, observation, and autotransplantation were also reported across studies [20,21,24].

Autotransplantation has been proposed as an alternative approach in selected cases, although supporting evidence remains limited and largely derived from small case series [24,25]. The coexistence of multiple management strategies underscores the absence of universally accepted prognostic criteria guiding treatment selection. This therapeutic variability further reflects the absence of a shared decision-making framework grounded in high-quality evidence.

Reported success rates for orthodontic traction ranged from 57% to 100% [20,21,22,23]; however, these figures must be interpreted with caution. Small sample sizes, retrospective selection, heterogeneous case severity, and inconsistent outcome definitions likely influenced reported estimates. In particular, selection bias may have led to preferential inclusion of cases with more favorable anatomical conditions, thereby potentially overestimating treatment success.

Importantly, success was generally defined as positional alignment within the dental arch, without standardized evaluation of periodontal health, pulp vitality, occlusal function, or long-term stability [20,21,22]. No study employed composite outcome measures, and none provided structured long-term follow-up data. This narrow definition of success may not adequately reflect the true biological and functional outcome of treatment. Because favorable outcomes were defined almost exclusively in positional terms, the reported success rates should be interpreted as an index of mechanical alignment rather than of biological or functional success; consequently, they are likely to overstate the true clinical predictability of the intervention, particularly with respect to periodontal integration, pulp vitality, and long-term occlusal stability. As a result, the true biological and functional predictability of orthodontic traction in mandibular cases remains uncertain.

Transmigration represents the most severe positional variant within the spectrum of mandibular impaction. It was originally defined by Javid (1985) and subsequently categorized into five positional patterns by Mupparapu (2002) [7,8]. While this classification provides a descriptive radiographic framework, none of the analytical studies included in the present review stratified treatment outcomes according to transmigration subtype. Most published data regarding transmigrated mandibular canines derive from isolated case reports or small case series, limiting the possibility of drawing reproducible prognostic conclusions [26,27,28,29,30,31].

Without positional stratification, it is not possible to determine whether orthodontic traction remains biologically feasible across all transmigration patterns or only in selected configurations. This represents a critical gap, as positional severity is likely one of the main determinants of treatment feasibility. In particular, although transmigration was reported in three of the four included studies (Table 4), none specified the number of transmigrated canines actually treated with traction or their corresponding Mupparapu subtype; consequently, the between-study variation in success rates (57–100%) cannot be attributed to specific positional patterns, such as crossing of the mandibular symphysis, and any such interpretation would remain speculative.

Methodological assessment further limits interpretability. All included studies were judged to have a serious overall risk of bias according to ROBINS-I, primarily due to uncontrolled confounding and selection bias. Across the four included studies, the mean age of the mandibular subgroups varied widely, from 12.3 to 19.2 years (Table 4), and no study adjusted outcomes for age or other baseline characteristics. This unadjusted heterogeneity is a principal contributor to the serious confounding rating (ROBINS-I domain 1) and may partly account for the divergent failure rates observed, since greater skeletal maturity, reduced periodontal ligament plasticity, and diminished eruptive potential in older patients are all biologically plausible determinants of less predictable traction. In the absence of individual patient-level data and age-stratified reporting, however, the direction and magnitude of this effect cannot be quantified, and age should be regarded as an uncontrolled confounder rather than a validated prognostic factor [21,32,33].

The GRADE assessment indicated very low certainty of evidence for all outcomes, reflecting risk of bias, imprecision related to the small cumulative sample (*n* = 42 treated teeth), and inconsistency in outcome reporting. Very low certainty implies that the true effect may be substantially different from currently reported estimates. Therefore, current evidence should be considered exploratory rather than confirmatory.

From a clinical perspective, surgical exposure followed by orthodontic traction may represent a viable option in carefully selected patients, particularly younger individuals and cases without extreme displacement. However, unlike maxillary impactions, evidence-based prognostic thresholds are not currently available for mandibular canines. Clinical decision-making must therefore rely on individualized assessment, three-dimensional radiographic evaluation, and clinician expertise rather than validated predictive algorithms [6,27,34].

This reinforces the need for cautious case selection and clear communication with patients regarding the uncertainty of outcomes.

Future research should prioritize multicenter collaboration, standardized outcome definitions, positional stratification—including transmigration subtypes—and structured long-term follow-up in order to move from empirically driven management toward more reproducible, protocol-oriented care. Only through such improvements will it be possible to establish reliable prognostic frameworks comparable to those available for maxillary canine impaction.

Surgical technique may represent another clinically relevant but insufficiently reported determinant of treatment outcome. In the broader impacted-canine literature, both open and closed surgical exposure techniques have been compared in terms of periodontal outcomes, treatment duration, patient discomfort, and esthetic results [35,36,37]. However, evidence is largely derived from maxillary canine impaction, and direct extrapolation to mandibular cases should be cautious. In mandibular canines, particularly those positioned lingually, closed exposure may be preferred because of limited surgical access and the reduced amount of keratinized tissue, although this recommendation remains primarily experience-based rather than supported by mandibular-specific comparative trials [38].

Orthodontic anchorage strategy may also influence treatment feasibility. One included study reported the use of temporary anchorage devices to assist traction of impacted mandibular canines [22]. Recent literature suggests that TAD-assisted mechanics may improve anchorage control, reduce unwanted dental side effects, and facilitate more controlled force application during impacted-canine traction [39,40]. Nevertheless, available evidence remains limited and mostly extrapolated from maxillary or mixed impacted-canine populations. Therefore, the role of TADs in mandibular canine impaction should be interpreted as a promising biomechanical adjunct rather than an evidence-based standard of care. Finally, the scarcity of evidence on rare presentations such as impacted mandibular canines also carries an educational cost, since trainees rarely encounter a representative caseload; here, emerging large language model-based tools may help convert isolated cases into reusable teaching resources [41].

### 4.1. Strengths and Limitations

Several limitations must be acknowledged when interpreting the present findings. First, the evidence base was quantitatively limited, as only four retrospective observational studies met the inclusion criteria, contributing a small cumulative sample of 42 mandibular canines treated with surgical exposure and orthodontic traction. Second, all included studies were judged to carry a serious overall risk of bias according to ROBINS-I—driven mainly by uncontrolled confounding and selection bias inherent to their retrospective design—and the certainty of evidence was rated as very low for every outcome according to GRADE. Third, the primary outcome (“success”) was defined inconsistently and, in most studies, was restricted to positional alignment within the dental arch, without standardized assessment of periodontal health, pulp vitality, occlusal function, or long-term stability; secondary outcomes were reported sparsely and were not uniformly stratified for the treated subgroup. Fourth, none of the included studies stratified outcomes by transmigration subtype, precluding any positional prognostic analysis. Finally, the prolonged recruitment periods of the included studies may have introduced further heterogeneity in diagnostic criteria, imaging modalities, and treatment protocols over time.

For these reasons, a quantitative synthesis (meta-analysis) was deliberately not undertaken. Pooling the available data would have been methodologically inappropriate given the substantial clinical and methodological heterogeneity across studies—specifically, the non-uniform operational definitions of success, the differing outcome-reporting formats, the variability in case severity and orthodontic mechanics (conventional fixed traction versus TAD-assisted traction), and the small, tooth-level sample sizes. Under these conditions, a pooled estimate would have conveyed a false impression of precision and could have obscured, rather than clarified, the true variability of the underlying evidence. A structured qualitative (narrative) synthesis was therefore adopted as the most transparent and scientifically defensible approach.

Notwithstanding these limitations, the present review has methodological strengths. It followed a prospectively registered protocol (PROSPERO CRD420261289636) in accordance with the PRISMA 2020 statement, restricted inclusion to analytical study designs providing extractable outcome data, and incorporated formal risk-of-bias (ROBINS-I) and certainty-of-evidence (GRADE) assessments—safeguards that were inconsistently applied in previous reviews on this topic.

### 4.2. Future Directions

Future clinical studies would benefit from investigating the surgical–orthodontic management of these cases as an integrated whole, considering several interrelated aspects, such as the exposure technique (open versus closed), the flap design and bonding/traction auxiliary employed, the timing between exposure and the onset of traction, the biomechanics and anchorage of traction, and the accompanying periodontal and endodontic response, so that their combined influence on treatment outcomes can be appreciated.

## 5. Conclusions

This systematic review highlights the limitations of the available evidence regarding the actual recovery of impacted mandibular canines following surgical exposure and orthodontic traction. Although alignment was reported in 31/42 teeth, small sample size, retrospective designs, and heterogeneity in case selection and outcome definitions limit the reliability of these findings. The high risk of bias and very low certainty of evidence make the true clinical effectiveness and long-term predictability uncertain. In contrast to maxillary canine impaction, validated prognostic thresholds and reproducible treatment protocols are lacking, and clinical management remains largely individualized. Further multicenter studies with standardized outcomes, including functional and periodontal parameters, as well as long-term follow-up and clear stratification of transmigration subtypes, are required to support evidence-based management.

## Figures and Tables

**Table 1:** Search strategy for each database.

Database	Search Strategy
Pubmed	(“Tooth, Impacted”[MeSH] OR impacted canine* OR ectopic canine* OR unerupted canine* OR retained canine*) AND (“Mandible”[MeSH] OR mandibular OR mandible OR lower jaw) AND (canine*)
Scopus	TITLE-ABS-KEY ((impacted OR impaction OR transmigration OR transmigrated) AND canine AND (mandibular OR mandible OR “lower jaw”))
Embase	(‘impacted canine’ OR ‘ectopic canine’ OR ‘unerupted canine’ OR ‘retained canine’ OR ‘transmigrated canine’) AND (mandibular OR ‘mandible’/exp OR mandible OR ‘lower jaw’/exp OR ‘lower jaw’) AND (‘canine’/exp OR canine)
Web of Science	(“impacted canine” OR “ectopic canine” OR “unerupted canine” OR “retained canine” OR “transmigrated canine”) AND (mandibular OR mandible OR “lower jaw”) AND canine
Cochrane Library	(mandibular canine) AND (impacted OR impaction OR transmigrated OR transmigration) in Title Abstract Keyword

Detailed descriptions of the search conducted in the five selected databases: PubMed, Scopus, Embase, Web of Science and the Cochrane Central Register of Controlled Trials (CENTRAL). The search was customized to each database.

**Table 2:** PICO question.

PICO	
**Participant**	Patients presenting with impacted mandibular canines, including transmigrated mandibular canines
**Intervention**	Surgical exposure with orthodontic traction aimed at alignment of the impacted mandibular canine into the dental arch
**Comparison**	Not mandatory. When available, comparisons between different surgical techniques, orthodontic mechanics, age groups, or positional characteristics were considered.
**Outcome**	*Primary outcome*: Successful alignment of the impacted mandibular canine in the dental arch. *Secondary outcomes*: Treatment duration; failure of orthodontic movement; need for extraction during treatment; root resorption; pulp vitality; ankylosis; periodontal complications; postoperative complications; and stability at follow-up when reported.

**Table 3:** General characteristics.

Study (Year)	Country	Study Design	Study Period (Years)	Sample Size (Patients)	Primary Aim
Aras et al., 2011 [20]	Turkey	Retrospective	1998–2006	21	To evaluate surgical–orthodontic management outcomes of impacted mandibular canines.
Sajnani & King, 2015 [21]	Hong Kong, China	Retrospective	1986–2013	74	To assess success rates and complications of different management strategies for impacted mandibular canines.
Sinko et al., 2016 [22]	Austria	Retrospective	2003–2015	56	To develop a clinical decision-making algorithm and evaluate management strategies for impacted and transmigrated lower canines.
Stabryła et al., 2021 [23]	Poland	Retrospective	NR	32	To compare treatment methods and clinical outcomes of impacted mandibular canines.

Sample size refers exclusively to patients with impacted mandibular canines included in the present analysis. NR = Not reported.

**Table 4:** Clinical characteristics of patients treated with surgical exposure and orthodontic traction.

**Study (Year)**	**Treated Canines (*n*)**	Mean Age * (Years)	**Transmigration Reported**	**Orthodontic Mechanics**
Aras et al. (2011) [20]	23	19.2	Yes	Conventional fixed traction
Sajnani & King (2015) [21]	7	12.3	Yes	Conventional fixed traction
Sinko et al. (2016) [22]	4	16.9	Yes	TAD-assisted traction
Stabryła et al. (2021) [23]	8	15.2	NS	Conventional fixed extrusion

Data refer to mandibular canines treated with surgical exposure and orthodontic traction. * Mean age reported for the mandibular subgroup when not specifically stratified for treated cases. Transmigration was recorded as reported by the original authors; treated-case numbers were not consistently specified. Orthodontic mechanics were classified according to each study’s description. NS = Not Specified.

**Table 5:** Clinical outcomes of impacted mandibular canines treated with surgical exposure and orthodontic traction.

**Study (Year)**	**Treated (*n*)**	**Successful (*n*)**	**Success Rate (%)**	**Failures (*n*)**	**Secondary Outcomes Reported**
Aras et al., 2011 [20]	23	16	69.6	7	Extractions; root resorption; ankylosis
Sajnani & King, 2015 [21]	7	4	57.1	3	Extractions
Sinko et al., 2016 [22]	4	3	75.0	1	Treatment discontinuation
Stabryła et al., 2021 [23]	8	8	100.0	0	No complications reported

Outcomes are reported at the tooth level. Success rates were calculated based on the number of treated mandibular canines. Secondary outcomes are presented as described in the original studies and were not uniformly stratified or quantified.

**Table 6:** Summary of the quality of evidence (GRADE) for the main outcomes evaluated.

**Outcome**	**Evidence Type**	**Starting Level**	**Risk of Bias**	**Inconsistency**	**Indirectness**	**Imprecision**	**Publication Bias**	**Upgrading Factors**	**Overall GRADE**
Successful orthodontic alignment [4 studies]	Observational studies	Low	Serious (−1): retrospective design, ROBINS-I overall serious risk	Serious (−1): success rates ranged from 57% to 100%	Not serious (0): directly addresses PICO	Serious (−1): small samples (*n* = 42), no pooled estimates	Undetected	None	Very low
Treatment duration [2 studies]	Observational studies	Low	Serious (−1): retrospective design, incomplete stratification	Serious (−1): non-standardized reporting	Not serious (0)	Very serious (−2): sparse data, inconsistent metrics	Undetected	None	Very low
Failure of eruption/lack of movement [3 studies]	Observational studies	Low	Serious (−1): confounding not controlled	Not serious (0): consistently reported mechanism of failure	Not serious (0)	Serious (−1): small numbers, tooth-level reporting	Undetected	None	Very low
Root resorption/ankylosis [1 study]	Observational studies	Low	Serious (−1): non-systematic assessment	Serious (−1): rarely reported	Not serious (0)	Very serious (−2): no consistent numerical data	Undetected	None	Very low
Periodontal outcomes [≤1 study]	Observational studies	Low	Serious (−1): retrospective, non-standardized evaluation	Serious (−1): heterogeneous or absent reporting	Not serious (0)	Very serious (−2): extremely limited data	Undetected	None	Very low
Outcomes in transmigrated canines [≤2 studies]	Observational studies	Low	Serious (−1): high confounding, selection bias	Serious (−1): clinical heterogeneity	Serious (−1): mixed populations, multimodal strategies	Very serious (−2): extremely small samples	Undetected	None	Very low
Long-term stability [no study reported follow-up data]	Observational studies	Low	Very serious (−2): absence of follow-up assessment	Not applicable	Serious (−1): outcome not directly assessed	Very serious (−2): no numerical data	Undetected	None	Very low

## Data Availability

No new data were created or analyzed in this study. All data supporting the findings are available within the article and were extracted from the publicly available published studies included in the systematic review.

## Supplementary Materials

The following supporting information can be downloaded at: https://www.mdpi.com/article/10.3390/dj14100632/s1. PRISMA 2020 checklist.
